# Construction of a circRNA-miRNA-mRNA Regulatory Network Reveals Potential Mechanism and Treatment Options for Osteosarcoma

**DOI:** 10.3389/fgene.2021.632359

**Published:** 2021-05-17

**Authors:** Yi He, Haiting Zhou, Wei Wang, Haoran Xu, Hao Cheng

**Affiliations:** ^1^Department of Orthopedics, Tongji Hospital, Tongji Medical College, Huazhong University of Science and Technology, Wuhan, China; ^2^Department of Oncology, Tongji Hospital, Tongji Medical College, Huazhong University of Science and Technology, Wuhan, China

**Keywords:** circRNA, osteosarcoma, network, GEO, TCGA

## Abstract

**Background:**

Osteosarcoma is a common malignant primary bone tumor in adolescents and children. Numerous studies have shown that circRNAs were involved in the proliferation and invasion of various tumors. However, the role of circRNAs in osteosarcoma remains unclear. Here, we aimed to explore the regulatory network among circRNA-miRNA-mRNA in osteosarcoma.

**Methods:**

The circRNA (GSE140256), microRNA (GSE28423), and mRNA (GSE99671) expression profiles of osteosarcoma were collected from the Gene Expression Omnibus (GEO) database. Differentially expressed circRNAs, miRNAs and mRNAs were identified. CircRNA-miRNA interactions and miRNA-mRNA interactions were determined by Circular RNA Interactome (CircInteractome) database and microRNA Data Integration Portal (mirDIP) database, respectively. Then, we constructed a regulatory network. Function enrichment analysis of miRNA and mRNA was performed by DIANA-miRPath v3.0 and Metascape database, respectively. mRNAs with significant prognostic value were identified based on expression profiles from The Cancer Genome Atlas (TCGA) database, and we constructed a subnetwork for them. To make the most of the network, we used the CLUE database to predict potential drugs for the treatment of osteosarcoma based on mRNA expression in the network. And we used the STITCH database to analyze and validate the interactions among these drugs and mRNAs, and to further screen for potential drugs.

**Results:**

A total of 9 circRNAs, 19 miRNAs, 67 mRNAs, 54 pairs of circRNA-miRNA interactions and 110 pairs of miRNA-mRNA interactions were identified. A circRNA-miRNA-mRNA network was constructed. Function enrichment analysis indicated that these miRNAs and mRNAs in the network were involved in the process of tumorigenesis and immune response. Among these mRNAs, STC2 and RASGRP2 with significantly prognostic value were identified, and we constructed a subnetwork for them. Based on mRNA expression in the network, three potential drugs, quinacridine, thalidomide and zonisamide, were screened for the treatment of osteosarcoma. Among them, quinacridine and thalidomide have been proved to have anti-tumor effects in previous studies, while zonisamide has not been reported. And a corresponding drug-protein interaction network was constructed.

**Conclusion:**

Overall, we constructed a circRNA-miRNA-mRNA regulatory network to investigate the possible mechanism in osteosarcoma, and predicted that quinacridine, thalidomide and zonisamide could be potential drugs for the treatment of osteosarcoma.

## Introduction

Osteosarcoma is a common malignant primary bone tumor in adolescents and children (Luetke et al., 2014). This tumor is most likely to happen in the metaphysis regions of long bones (Cortini et al., 2017). Medical advances have significantly improved the survival rate for osteosarcoma. However, early screening and diagnosis of osteosarcoma are arduous due to the lack of adequate diagnostic markers (Li and Wang, 2020). Metastasis and drug resistance also worsened the prognosis (Mirabello et al., 2009; Harrison et al., 2018). It is urgent to figure out the underlying pathogenesis of osteosarcoma, and to discover potential targets for earlier diagnosis and potential drugs for better treatment.

CircRNA is a new class of endogenous and regulatory non-coding RNA with a covalent closed-loop structure. CircRNAs were discovered as early as the 1990s (Nigro et al., 1991). However, due to limitations in knowledge and technology at that time, they were not be fully investigated and thought to be less abundant *in vivo* due to splicing errors (Jeck and Sharpless, 2014). With the development of high-throughput sequencing technology, thousands of circRNAs have been recognized (Zhang et al., 2014). Numerous studies have shown that circRNAs were involved in tumor proliferation and invasion, and could become molecular markers of various tumors (Cheng et al., 2019; Liu et al., 2019; Li et al., 2020).

**FIGURE 1:**
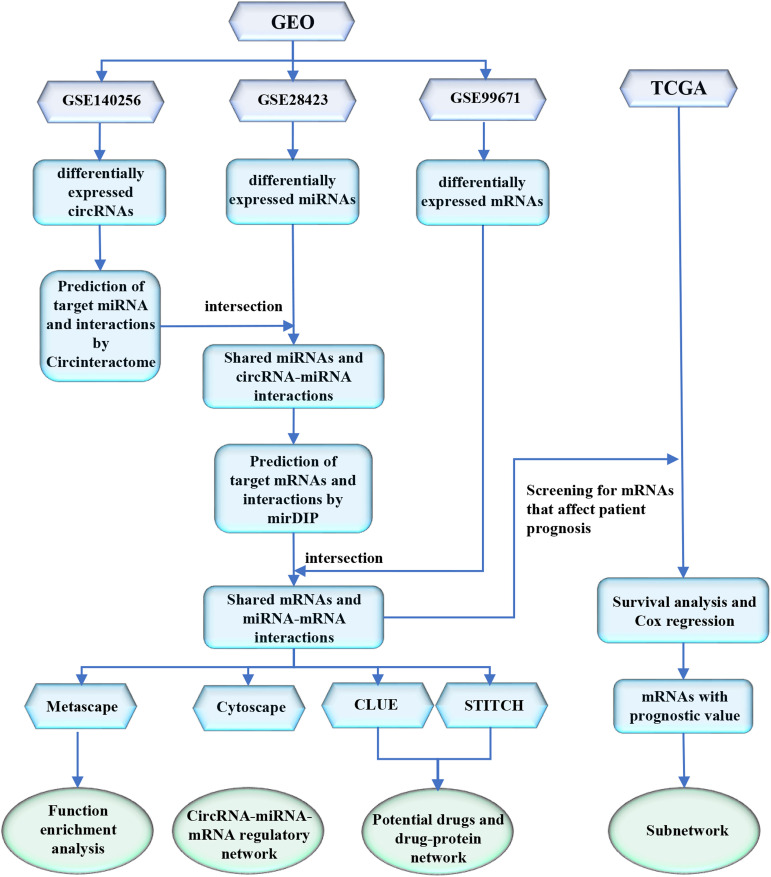
A flow chart of the analytical process.

In the present study, we collected the expression profiles of circRNAs (GSE140256), miRNAs (GSE28423), and mRNAs (GSE99671) of osteosarcoma from GEO database. We also collected another mRNA expression profiles with survival information from TCGA database. The process flow chart is shown in Figure 1. Using expression profiles from GEO to perform differential expression analysis and targets prediction, we determined the circRNA-miRNA interactions and the miRNA-mRNA interactions, and finally constructed a circRNA-miRNA-mRNA regulatory network. Using expression profiles from the TCGA database, we identified mRNAs with significant prognostic value. Furthermore, we performed functional enrichment analysis to reveal the potential mechanism of osteosarcoma. Particularly, we predicted potential drugs for the treatment of osteosarcoma, which may provide new insight into osteosarcoma treatment.

## Materials and Methods

### Data Sets

Gene Expression Omnibus (GEO)^1^ is a public functional genomics data repository that helps researchers query and download experiments and curated gene expression profiles. Three datasets (GSE140256, GSE28423, and GSE99671) from the GEO database were collected in our study. GSE140256 is a microarray chip dataset of circRNAs containing 3 primary osteosarcoma tissues and 3 adjacent tissues, and we used this dataset to screen for differentially expressed circRNAs. GSE28423 is a microarray chip dataset of miRNAs containing samples from 19 human osteosarcoma cell lines and 4 normal bones, which was used to screen for differentially expressed miRNAs. GSE99671 is a dataset of expression profiling by high throughput sequencing, containing 18 osteosarcoma samples and 18 corresponding normal bone samples, which was used to screen for differentially expressed mRNAs.

The Cancer Genome Atlas (TCGA)^2^ is a landmark cancer genomics program demonstrating the genomic alterations associated with 33 cancer types. 88 osteosarcoma samples were obtained from the database. After data processing, 3 samples were removed due to incomplete survival information or follow-up time less than 30 days (Subramanian et al., 2005). Finally, 85 samples from TCGA with complete clinical information were included in our study for survival analysis.

### The Acquisition of Differential Expression of circRNAs, miRNAs, and mRNAs

The expression profiles of GSE140256 and GSE28423 were normalized by limma package, and that of GSE99671 was normalized with TMM (Trimmed Mean of M values) methods by edgeR package (Robinson et al., 2010). Then differentially expressed circRNAs, miRNAs and mRNAs were obtained from GSE140256, GSE28423, and GSE99671, respectively. Differentially expressed circRNAs, miRNAs, mRNAs were defined as adjusted *P* < 0.05 and | log2 fold-change (FC)| > 1.5 (Wang et al., 2019).

### Prediction of circRNA Targeted miRNAs

Circular RNA Interactome (CircInteractome)^3^ is an online tool based on 109 datasets of RNA-binding proteins (RBPs) and queries circRNAs for RNA-binding sites, which enables to predict RNA-binding proteins and miRNAs on circRNAs (Dudekula et al., 2016). CircRNA-targeted miRNAs were predicted by the database, from which we also acquired the interactions among circRNAs and miRNAs. Then these predicted miRNAs were intersected with differentially expressed miRNAs from GSE28423 dataset to obtained shared miRNAs and their interactions with circRNAs.

### Prediction of miRNA Targeted mRNAs

MicroRNA Data Integration Portal (mirDIP)^4^ is a web tool to predict miRNA-mRNA interactions which integrated information on human miRNA targeted mRNAs from 30 databases (Tokar et al., 2018). We uploaded the shared miRNAs obtained in the previous step to the database to acquire the predicted mRNAs and miRNA-mRNA interactions. In order to make the prediction more accurate, the score class for the confidence was set at the top 1%. After that, these predicted mRNAs were selected to intersect with the differentially expressed mRNAs from GSE99671 dataset to obtained shared mRNAs and their interactions with miRNAs.

### Functional Enrichment Analysis

DIANA-miRPath v3.0^5^ is an online-server providing evaluation of the regulatory role of miRNAs (Vlachos et al., 2015). We used the database to analyze the function of these miRNAs. The Metascape database^6^ is a powerful gene function annotation analysis tool (Zhou et al., 2019). Gene enrichment analysis was performed on Metascape database. The min overlap was set at 3, which required enriched terms to include ≥ 3 candidates. The min enrichment was set at 1.5, which indicated at least 1.5 times more given pathway members are found in uploaded gene list compared to what would have been expected by chance. The *P*-value was set at < 0.01.

### Survival Analysis in TCGA Profiles

Since the GEO datasets did not contain survival information, we estimated the prognostic value of shared mRNAs using Kaplan-Meier method and Cox regression analysis based on expression profiles from TCGA dataset. The expression profiles of osteosarcoma from TCGA database were transformed into TPM format for normalization. The threshold of statistical significance of log-rank test was set at *P*-value < 0.05.

### Construction of circRNA-miRNA-mRNA Network

In the previous steps, we obtained differentially expressed circRNAs, shared miRNAs, shared mRNAs and their interactions. Then we used their interactions to construct a circRNA-miRNA-mRNA regulatory network and we visualized it by using Cytoscape software (Version 3.8.0).

### Prediction of Potential Drugs and the Construction of Drug-Protein Interaction Network

To make the most of the established network, we performed prediction of potential therapeutic drugs. The Connectivity Map Linked User Environment (CLUE)^7^ is the world’s largest perturbation-driven gene expression dataset (Subramanian et al., 2017). We used it to identify compounds whose administration to cancer cells resulted in an opposite expression profile of these mRNAs in the network. The database would calculate a score (−100∼100) for each compound within database, called connectivity score, based on the expression of queried mRNAs. In particular, a negative score would indicate that the compound was antagonistic to the expression of queried mRNAs, that is, the expression of queried mRNAs was reduced by treatment of the compound. Since the mRNAs we queried were associated with tumor development, reversing their expression by the drugs might inhibit tumor growth. We upload 67 mRNAs from the network to the CLUE database and collated the results to filter out compounds with the connectivity score < −85 (Chen Y.T. et al., 2019).

Search Tool for Interacting Chemicals (STITCH)^8^ is a database for predicting interactions between chemical substances and proteins (Szklarczyk et al., 2016). To further screen the compounds, we looked for compounds that interacted with the proteins, which were coded by mRNAs from our network, by using STITCH database. We set the interaction score > 0.7, which indicated a high confidence. Predicted functional partners < 5, which indicated less than 5 other predicted proteins would be involved in the drug and protein interactions. The results were visualized by a drug-protein interaction network by using Cytoscape software.

## Results

### The Acquisition of Differential Expression of circRNAs, miRNAs, and mRNAs

**FIGURE 2:**
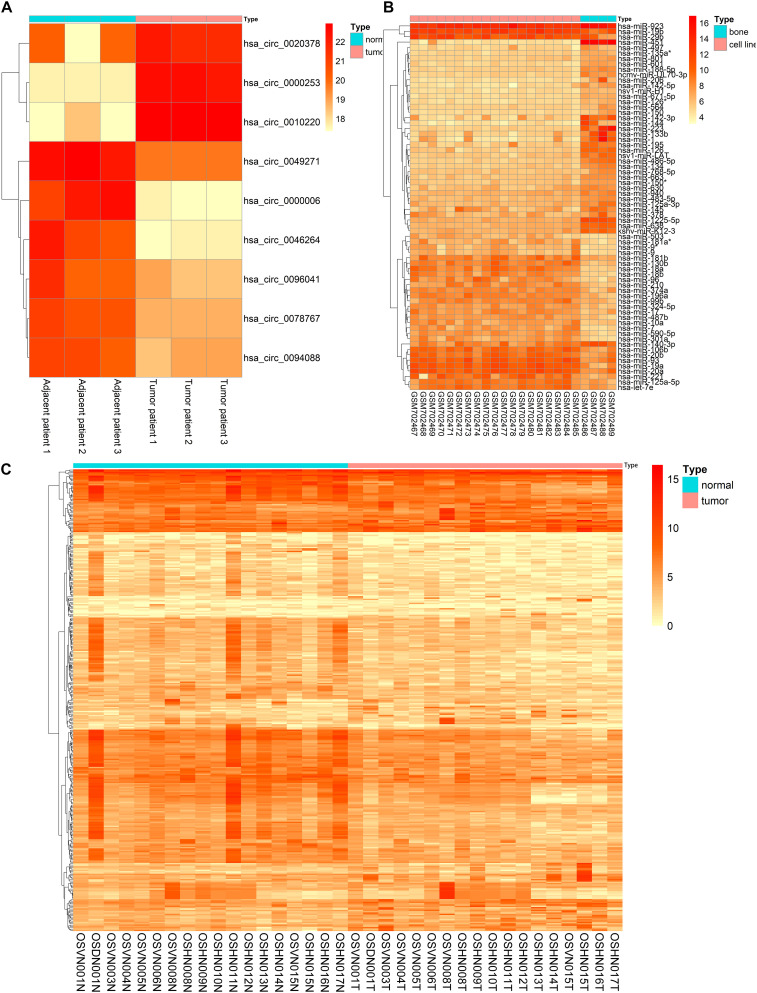
The heatmaps of differentially expressed circRNAs **(A)**, miRNAs **(B)**, and mRNAs **(C)**.

The basic information of three microarray datasets (GSE140256, GSE28423, and GSE99671) was listed in Table 1. In GSE140256 dataset, 9 differentially expressed circRNAs were screened out. The basic information for 9 circRNAs was showed in Table 2. Among them, hsa_circ_0000253, hsa_circ_0010220, and hsa_circ_0020378 were up-regulated and might be tumor promotors in osteosarcoma. While hsa_circ_0049271, has_circ_0000006, hsa_circ_0078767, hsa_circ_0046264, hsa_circ_0094088, and hsa_circ_0096041 were down-regulated and might be tumor inhibitors in osteosarcoma. In GSE28423 dataset and GSE99671 dataset, we screened out 68 differentially expressed miRNAs and 346 differentially expressed mRNAs, respectively. The heatmaps of differentially expressed circRNAs, miRNAs and mRNAs were shown in Figure 2.

**TABLE 1:** Basic information of the three microarray datasets from GEO and an RNAseq dataset from TCGA.

RNA	Dataset	Platform	Sample size (Normal/Tumor)
circRNA	GSE140256	GPL27741	3/3
miRNA	GSE28423	GPL8227	4/19
mRNA	GSE99671	GPL20148	18/18
mRNA	TCGA	–	0/85

**TABLE 2:** Basic characteristics of nine circRNAs.

CircRNA	Position	Strand	Genomic length	Best transcript	Gene symbol	Regulation
hsa_circ_0000253	chr10:97999787-97999925	−	138	NM_013314	BLNK	Up
hsa_circ_0010220	chr1:17907047-18024370	+	117323	NM_018125	ARHGEF10L	Up
hsa_circ_0049271	chr19:10610070-10610756	−	686	NM_203500	KEAP1	Down
hsa_circ_0000006	chr1:1601102-1666274	−	65172	NM_001110781	SLC35E2B	Down
hsa_circ_0078767	chr6:170615843-170639638	+	23795	NM_032448	FAM120B	Down
hsa_circ_0046264	chr17:79813017-79817263	−	4246	NM_000918	P4HB	Down
hsa_circ_0094088	chr10:7318853-7407477	−	88624	NM_001029880	SFMBT2	Down
hsa_circ_0096041	chr11:61615630-61616333	+	703	ENST00000278840.4	FADS2	Down
hsa_circ_0020378	chr10:128594022-128926028	+	332006	NM_001380	DOCK1	Up

### Identification of circRNA–miRNA Interactions

Using CircInteractome database, we identified 313 targeted miRNAs for these 9 circRNAs, and there were 951 pairs of circRNA-miRNA interactions among them. Using these 313 predicted miRNAs to intersect with the 68 differentially expressed miRNAs acquired from GSE28423 dataset, we finally obtained 19 shared miRNAs. One circRNA had no corresponding targeted miRNA after intersections. Thus, we obtained 54 pairs of interactions among 8 circRNAs and 19 miRNAs.

### Identification of miRNA–mRNA Interactions

We uploaded the 19 shared miRNAs obtained in the previous step to mirDIP database (Tokar et al., 2018), and the score class for the confidence was set at the top 1% for more accurate prediction. Then we acquired 6827 mRNAs targeted by these 19 miRNAs. And there were 13431 pairs of interactions among them. These 6827 mRNAs were then intersected with the differentially expressed mRNAs obtained from the differential analysis of GSE99671 dataset, and finally we identified 67 shared mRNAs and 110 pairs of miRNA-mRNA interactions.

### Function Enrichment Analysis

**FIGURE 3:**
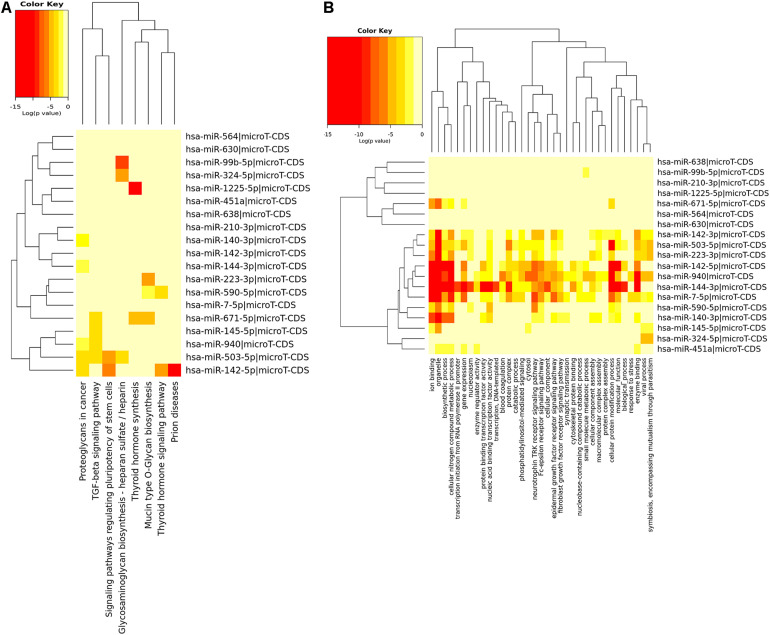
KEGG pathway enrichment analysis **(A)** and GO enrichment analysis **(B)** of 19 shared miRNAs based on DIANA-miRPath.

Using DIANA-miRPath database, we found that these shared miRNAs were involved in TGF-beta signaling pathway, proteoglycans in cancer, epidermal growth factor receptor signaling pathway, fibroblast growth factor receptor signaling pathway and other pathways related to tumorigenesis, as shown in Figure 3.

**FIGURE 4:**
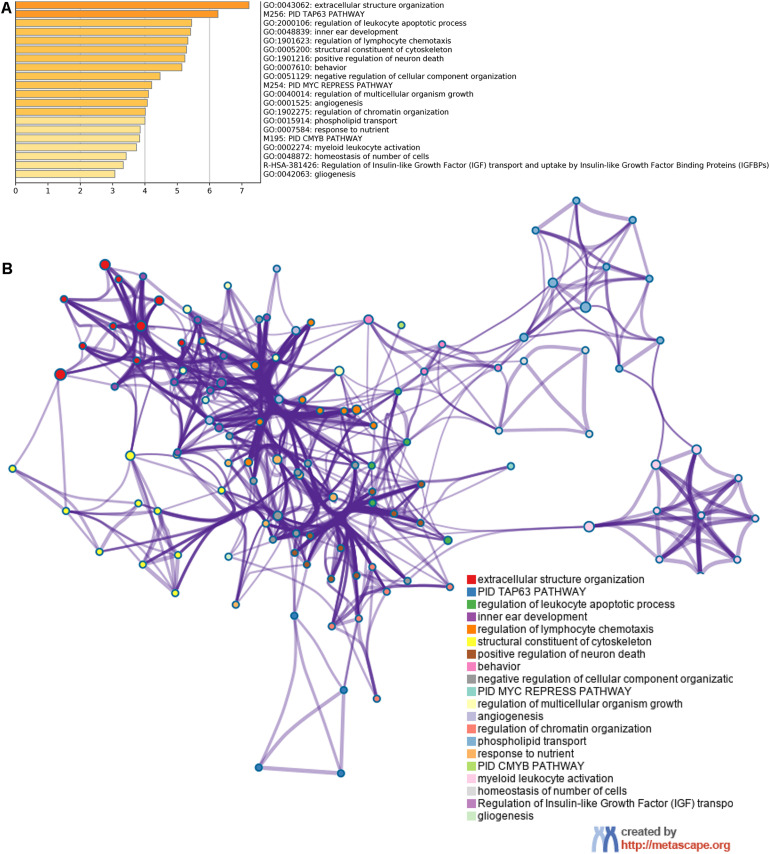
Function enrichment analysis of 67 shared mRNAs. **(A)** The bar graph of top clusters with their representative enriched terms and **(B)** a network of enriched terms, colored by cluster-ID, where nodes that share the same cluster-ID are typically close to each other.

Using Metascape database, we found that these shared mRNAs were predominantly enriched in cancer and immune-related functional activities and pathways (Figure 4). For Gene Ontology (GO) terms, mRNAs were enriched in extracellular structure organization, regulation of leukocyte apoptotic process, regulation of lymphocyte chemotaxis, negative regulation of cellular component organization, angiogenesis, myeloid leukocyte activation, homeostasis of number of cells and gliogenesis. For Canonical Pathways, mRNAs were enriched in TAP63 pathway, MYC repress pathway and CMYB pathway. For Reactome Gene Sets, mRNAs were enriched in Regulation of Insulin-like Growth Factor (IGF) transport and uptake by Insulin-like Growth Factor Binding Proteins (IGFBPs).

### Construction of a circRNA-miRNA-mRNA Network

**FIGURE 5:**
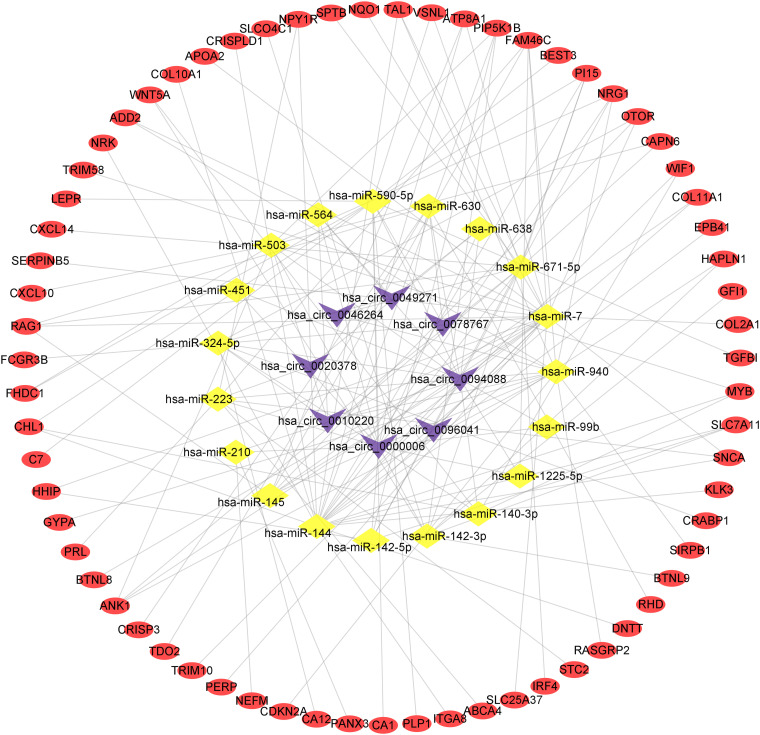
The circRNA-miRNA-mRNA regulatory network. The purple V represents circRNAs, the yellow diamond represents miRNAs and the red oval represents mRNAs.

To present the relationship among circRNAs, miRNAs, and mRNAs, we constructed a circRNA-miRNA-mRNA regulatory network based on the interactions among these transcripts and visualized it using Cytoscape software. The result was showed in Figure 5.

### Survival Analysis in TCGA Profiles

**FIGURE 6:**
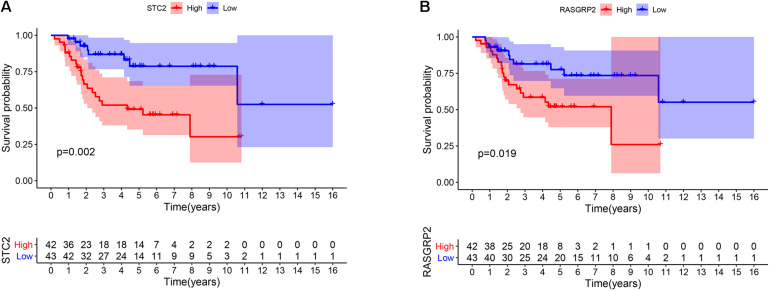
The Kaplan-Meier survival curve of **(A)** STC2 and **(B)** RASGRP2.

**FIGURE 7:**
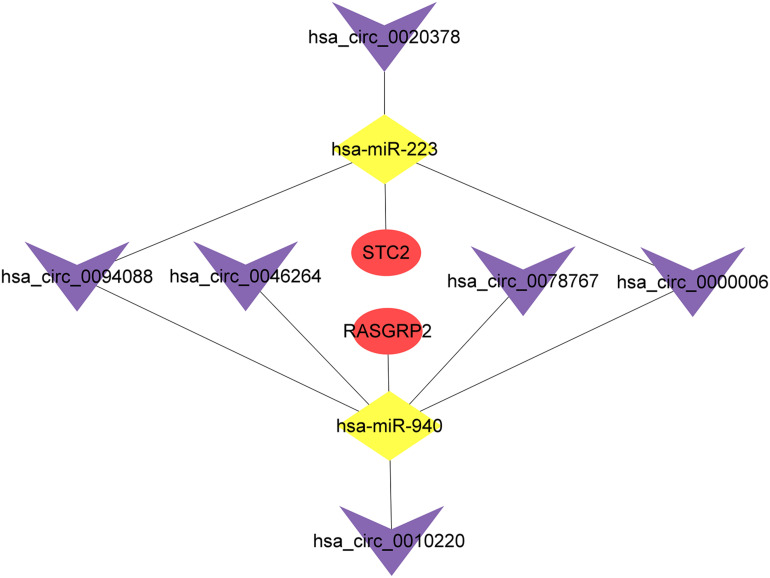
The circRNA-miRNA-mRNA subnetwork based on mRNAs screened out with prognostic value. The purple V represents circRNAs, the yellow diamond represents miRNAs and the red oval represents mRNAs.

To explore the prognostic value of 67 shared mRNAs in the network, we performed Kaplan-Meier survival analysis and Cox regression analysis based on profiles from TCGA database. Ultimately, two mRNAs (STC2 and RASGRP2) with significantly prognostic value were screened out, as shown in Table 3 and Figure 6. High expression of STC2 and RASGRP2 were correlated with poor prognosis of osteosarcoma. Stanniocalcin 2 (STC2) and Ras guanyl nucleotide releasing protein 2 (RASGRP2) were reported to be involved in many human malignancies (Tamura et al., 2009; Yokobori et al., 2010; Mele et al., 2018; Zhang et al., 2019). In order to show the regulatory mechanisms of these two mRNAs more clearly, we established a subnetwork contained these two mRNAs and associated miRNAs (hsa-miR-940 and hsa-miR-223) and circRNAs (hsa_circ_0010220, hsa_circ_0000006, hsa_circ_0078767, hsa_circ_0046264, hsa_circ_0094088, and hsa_circ_0020378) (Figure 7).

**TABLE 3:** Survival analysis according to the Kaplan-Meier method and the Cox method for 67 shared mRNAs in TCGA profile.

mRNA	KM	Hazard ratio (HR)	HR.95L	HR.95H	*P*-value
RASGRP2	0.01941	1.05537	1.01768	1.09446	0.00368
STC2	0.00174	1.01455	1.00390	1.02531	0.00730

### Identification of Potential Drugs and Construction of Drug-Protein Interaction Network

We uploaded 67 shared mRNAs to CLUE database. Based on the criteria of connectivity score < −85, we obtained 26 kinds of candidate compounds (drugs).

**FIGURE 8:**
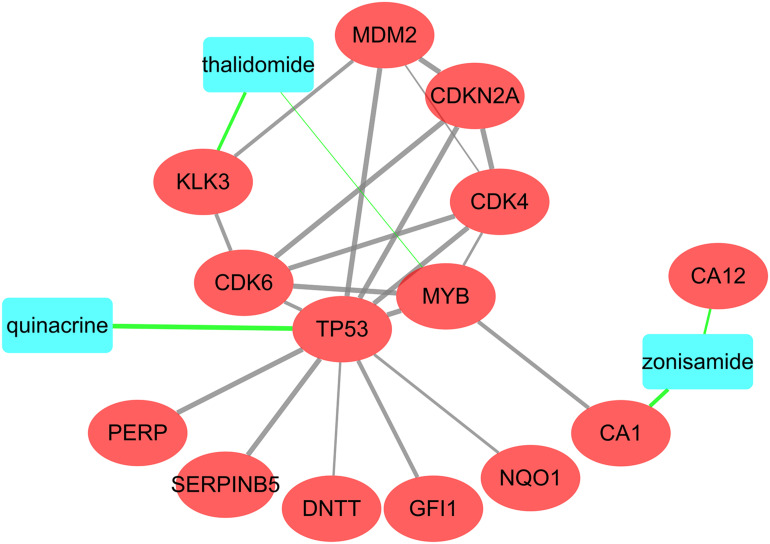
Interactions among the three potential drugs and proteins coded by mRNAs. The blue rectangle represents the drugs and the red oval represents the proteins coded by shared mRNAs and other predicted proteins involved in the interaction.

In order to further screen the compounds, we analyzed the interaction relationships among these candidate compounds and mRNAs. We uploaded the 26 candidate compounds and 67 mRNAs to STITCH database, and constructed a drug-protein network to visualize their interactions (Figure 8). Finally, only three compounds, quinacridine, thalidomide and zonisamide, were screened out. The information of these drugs was shown in Table 4.

**TABLE 4:** Potential drugs identified by CLUE and STITCH database for osteosarcoma.

Compounds	Description	Score
Quinacridine (Mepacrine)	Cytokine production inhibitor	−88.97
Thalidomide	TNF production inhibitor	−86.06
Zonisamide	Sodium channel blocker	−98.03

## Discussion

CircRNAs can act as miRNA sponges and bind to miRNA, relieving the inhibitory effect of miRNAs on their target mRNAs and regulating the expression of target mRNAs, which is known as the competitive endogenous RNA (ceRNA) mechanism (Qi et al., 2015). By interacting with cancer-associated miRNAs, circRNAs play an important regulatory role in cancer (Meng et al., 2017).

To investigate the potential role of circRNAs in osteosarcoma, we constructed a circRNA-miRNA-mRNA regulatory network using bioinformatics predictions combined with differential expression data. This network contained the interactions of 8 circRNAs, 19 miRNAs, and 67 mRNAs. Among these circRNAs, hsa_circ_0078767 was reported to suppress non-small-cell lung cancer by modulating RASSF1A expression via sponging miR-330-3p (Chen T. et al., 2019). While hsa_circ_0046264 was reported to have different effects in different tumors. Hsa_circ_0046264 could up-regulate BRCA2 by targeting miR-1245 to induce apoptosis and inhibit cell proliferation and invasion in lung cancer cells (Yang et al., 2018). However, Liu et al. proposed the opposite conclusion that hsa_circ_0046264 was remarkably up-regulated in lung cancer patients, enhancing tumor growth, invasion, metastasis, and chemotherapy resistance (Liu et al., 2020). Hsa_circ_0049271 was down-regulated in non-small cell lung cancer, which was similar to the down-regulation we found in osteosarcoma (Li et al., 2021). The other 6 circRNAs had not been studied yet, which need further investigation, and they have the potential to be reliable biomarkers and therapeutic targets.

Function enrichment analysis showed that these miRNAs and mRNAs were mainly involved in immune and inflammatory response, angiogenesis, and some common biological processes involved in tumorigenesis. Among them, MYC mediated transcriptional amplification through super-enhancers is an essential hallmark of cancer (Kress et al., 2015). MYC was demonstrated to be related to progression and poor prognosis in osteosarcoma. Besides, MYC could be suppressed by super-enhancer inhibitors to effectively inhibit growth, migration, and invasion of osteosarcoma cells (Chen et al., 2018). Therefore, targeting MYC/super-enhancer axis represents a promising treatment strategy for patients with osteosarcoma. According to animal model systems, bone tumors’ growth and metastasis depend on new blood vessel development, which is also known as angiogenesis (Gorlick et al., 2003). Vascular endothelial growth factor (VEGF) is a pivotal tumor-derived angiogenic factor with multiple biological functions, which constitute the most vital signaling pathways in tumor angiogenesis (Quan and Choong, 2006). The expression of VEGF has been considered as an important prognostic biomarker evaluating the angiogenesis in osteosarcoma (DuBois and Demetri, 2007). P63 is a member of the p53 family, which encodes the isoforms TAp63 and ΔNp63 (Ram Kumar et al., 2014). TAp63 functions as a tumor suppressor by regulating senescence through p53-independent pathways. The ability of TAp63 to trigger senescence and inhibit tumorigenesis without regard to p53 status, which identified TAp63 as a promising target of anti-cancer treatment for malignancies with compromised p53 (Guo et al., 2009).

Among these 67 mRNAs, we found that two mRNAs (STC2 and RASGRP2) were significantly related to overall survival. STC2 and RASGRP2 were associated with tumor development. It was reported that STC2 could promote head and neck squamous cell carcinoma metastasis by modulating the PI3K/AKT/snail signaling pathway (Yang et al., 2017). High expression of STC2 could also enhance the ability of migration and invasion for nasopharyngeal cancer cells after radiotherapy (He et al., 2019). In osteosarcoma, STC2 was reported to promote the proliferation, invasion and migration of osteosarcoma cells by enhancing the glycolysis (Yu et al., 2021). RASGRP2 is a guanine nucleotide exchange factor, which is well known to target to Rap1 mainly (Stone, 2011). RASGRP2 could inhibit apoptosis by activating Rap1 to inhibit tumor necrosis factor (TNF)-induced reactive oxygen species (ROS) production (Sato et al., 2019). This inhibition of apoptosis is likely to play an important role in tumor development as well. It has also been reported that calcium-sensitive RASGRP2 could promote chronic lymphocytic leukemia cell metastasis through activation of Rap1 (Mele et al., 2018). In addition, it was reported that African American enriched splice variants of PIK3CD, FGFR3, TSC2 and RASGRP2 have greater oncogenic potential compared to the corresponding European American expression variants, suggesting the oncogenic effect of RASGRP2 (Wang et al., 2017). Many studies supported the anti-tumor effects of hsa-miR-940. It was reported that hsa-miR-940 could inhibit the growth of hepatocellular carcinoma by targeting SPOCK1 (Li et al., 2019). MiR-940 was also reported to inhibit the progression of NSCLC by targeting FAM83F (Gu et al., 2018). However, no study has reported its role in osteosarcoma. But it was reported that cancer cells could secrete hsa-miR-940 to the bone microenvironment and induce an osteogenic phenotype by targeting ARHGAP1 and FAM134A (Hashimoto et al., 2018). It is well known that most osteosarcomas are osteolytic phenotypes. In our study, hsa-miR-940 was down-regulated, suggesting that it may play a tumor suppressor role, and this may also explain why osteosarcomas predominantly show an osteolytic rather than an osteogenic phenotype. Hsa-miR-223 was demonstrated to act as an oncogene in gastric cancer by targeting FBXW7/hCdc4 to regulate cell apoptosis, proliferation and invasion (Li et al., 2012). Hsa-miR-223 was also reported to work as a tumor-promotor in vulvar carcinoma by TP63 suppression (de Melo Maia et al., 2016). However, hsa-miR-223 was down-regulated in our study. We speculated mechanisms of the miRNAs vary among different malignancies. Thus, their regulatory relationships deserve further study.

To make the most of the network, we used it to explore potential compounds or drugs with reliable therapeutic effects for osteosarcoma. By using CLUE database and STITCH database, we screened out three candidate drugs, quinacridine, thalidomide and zonisamide. Quinacridine, also known as mepacrine, was originally used as an anti-malarial agent. But recently, researchers have found that quinacridine could intercalate into DNA, impact nuclear proteins, inhibit the NFκB pathway and induce p53 expression to exhibit cytotoxicity on cancer cells (Oien et al., 2021). Pellerano et al. (2017) found that quinacridine analogs could bind CDK2/Cyclin A and inhibit its kinase activity to inhibit cancer cell proliferation, and promote accumulation of cells in S phase and G2. Hounsou et al. (2007) found that quinacridine had quadruplex binding properties and was able to target and interact with G-tetrads of the terminal part of the telomere, which made quinacridine a potentially powerful candidate for anti-cancer strategy. In addition, quinacridine was reported to enhance and restore the sensitivity of cisplatin in some cancer, which provided a new possible strategy for chemotherapy combinations (Friedman et al., 2007; Wang et al., 2010). Moreover, quinacridine has been applied in clinical practice for a long time with fewer side effect (Sokal et al., 2008). However, there is no study related to this drug in osteosarcoma. Currently, chemoresistance is a major obstacle in the treatment of osteosarcoma. Thus, quinacridine is a drug with great potential for application in osteosarcoma. Further trials are needed to evaluate its potential application. Thalidomide is a drug with anti-inflammatory and immunomodulatory properties. It was originally used to treat respiratory infections and to relieve morning sickness in pregnant women. However, it was withdrawn as it was found to be teratogenic (Sherbet, 2015). But thalidomide was reported to inhibit tumor cell proliferation, angiogenesis and induce apoptosis recently (Mercurio et al., 2017). Thalidomide has been also used to treat some malignancies, such as refractory multiple myeloma, prostate cancer and malignant glioma in the past (Singhal et al., 1999; Marx et al., 2001; Drake et al., 2003). And it was reported that the use of thalidomide and its analogs significantly improved the prognosis of patients with multiple myeloma (Holstein and McCarthy, 2017). A meta-analysis showed that thalidomide combined with transcatheter arterial chemoembolization (TACE) had better clinical outcomes and tolerable adverse events in patients with primary liver cancer compared to TACE alone (Gao et al., 2016). Thalidomide has also been verified to cause apoptosis in osteosarcoma cell lines (Zhu et al., 2016). A case report reported celecoxib combined with thalidomide in the treatment of refractory osteosarcoma, and achieved favorable outcome (Tsai et al., 2005). These results suggested thalidomide has potential to be an anti-tumor drug for osteosarcoma. However, there is still a lack of extensive clinical trials to support its use in osteosarcoma. Zonisamide is primarily used to treat epilepsy. No study has reported its effect in cancer. Further research is needed on whether it can be used in the treatment of osteosarcoma. The drug-protein network we constructed might contribute to the further study of these drugs.

To date, a previous study has constructed a circRNA-miRNA-mRNA regulatory network, but the circRNA microarray was based on 7 osteosarcoma cell lines and 1 normal bone cell, and the miRNA microarray was based on serum of osteosarcoma patients, not tumor tissues or cells (Qiu et al., 2020). The circRNA microarray in our study included 3 osteosarcoma tumor tissues and paired normal tissues, which were more comparable, and the expression data of miRNA microarray was from osteosarcoma cell lines. Therefore, our study can provide some new insights into the circRNA-miRNA-mRNA network regulatory network of osteosarcoma. Moreover, we predicted potential drugs that might be effective in the treatment of osteosarcoma. It will be helpful in providing new perspectives in the treatment of osteosarcoma. However, several limitations should be considered. First, our study was based a range of bioinformatics analysis methods and online databases. Next, when performing target and drug predictions, we have chosen the latest versions of data, the latest algorithms and a high degree of confidence level, but there may still be some inevitable random errors and selection bias.

## Data Availability Statement

Publicly available datasets were analyzed in this study. This data can be found here: https://www.ncbi.nlm.nih.gov/geo/ and https://portal.gdc.cancer.gov/.

## Author Contributions

HC and YH performed the conception and design of this manuscript. YH and HZ provided useful suggestions in methodology. YH and WW performed data analysis and prepared the figures. HZ and HX drafted and revised the manuscript. All authors read and approved the final manuscript.

## Conflict of Interest

The authors declare that the research was conducted in the absence of any commercial or financial relationships that could be construed as a potential conflict of interest.

## Footnotes

1 https://www.ncbi.nlm.nih.gov/geo/

2 https://portal.gdc.cancer.gov/

3 https://circinteractome.irp.nia.nih.gov/

4 http://ophid.utoronto.ca/mirDIP/index.jsp

5 http://snf-515788.vm.okeanos.grnet.gr/

6 https://metascape.org/gp/index.html

7 https://clue.io/

8 http://stitch.embl.de/

## Abbreviations

TCGA: The Cancer Genome Atlas
GEO: Gene Expression Omnibus
CircInteractome: Circular RNA Interactome
MirDIP: MicroRNA Data Integration Portal
RBP: RNA-binding protein
GO: Gene Ontology
CeRNA: Competitive endogenous RNA
ROS: Reactive oxygen species
STC2: Stanniocalcin 2
RASGRP2: Ras guanyl nucleotide releasing protein 2.
